# The efficacy of transcranial direct current stimulation and transcranial magnetic stimulation for chronic orofacial pain: A systematic review

**DOI:** 10.1371/journal.pone.0221110

**Published:** 2019-08-15

**Authors:** Natália R. Ferreira, Ygor N. Junqueira, Nathália B. Corrêa, Estevão O. Fonseca, Nathália B. M. Brito, Thayná A. Menezes, Márcio Magini, Tatiana K. S. Fidalgo, Daniele M. T. P. Ferreira, Rodrigo L. de Lima, Antônio C. Carvalho, Marcos F. DosSantos

**Affiliations:** 1 Laboratório de Morfogênese Celular (LMC), Instituto de Ciências Biomédicas, Universidade Federal do Rio de Janeiro, Rio de Janeiro, Brazil; 2 Programa de Pós-Graduação em Radiologia, Faculdade de Medicina, Universidade Federal do Rio de Janeiro, Rio de Janeiro, Brazil; 3 Campus Macaé, Universidade Federal do Rio de Janeiro, Macaé, Rio de Janeiro, Brazil; 4 Laboratório de Análise e Processamento de Sinais, Universidade Federal do Rio de Janeiro, Campus Macaé, Macaé, Rio de Janeiro, Brazil; 5 Departamento de Odontologia Preventiva e Comunitária, Universidade do Estado do Rio de Janeiro, Rio de Janeiro, Brazil; 6 Biblioteca do Centro de Ciências da Saúde, Universidade Federal do Rio de Janeiro, Rio de Janeiro, Brazil; 7 Departamento de Ortodontia e Odontopediatria, Universidade Federal do Rio de Janeiro, Rio de Janeiro, Brazil; University of Mississippi Medical Center, UNITED STATES

## Abstract

**Background:**

Transcranial Direct Current Stimulation (tDCS) and Transcranial Magnetic Stimulation (TMS) have been described as promising alternatives to treat different pain syndromes. This study evaluated the effects of TMS and tDCS in the treatment of chronic orofacial pain, through a systematic review.

**Methods:**

An electronic search was performed in major databases: MEDLINE, Scopus, Web of Science, Cochrane, Embase, LILACS, BBO, Open Gray and CINAHL. The eligibility criteria comprised randomized clinical trials (RCTs) that applied TMS or tDCS to treat chronic orofacial pain. The variables analyzed were pain, functional limitation, quality of life, tolerance to treatment, somatosensory changes, and adverse effects. The risk of bias was assessed through the Cochrane Collaboration tool, and the certainty of evidence was evaluated through GRADE. The protocol was registered in the PROSPERO database (CRD42018090774).

**Results:**

The electronic search resulted in 636 studies. Thereafter, the eligibility criteria were applied and the duplicates removed, resulting in eight RCTs (four TMS and four tDCS). The findings of these studies suggest that rTMS applied to the Motor cortex (M1), the dorsolateral prefrontal cortex (DLPFC) and the secondary somatosensory cortex (S2) provide adequate orofacial pain relief. Two studies reported significant pain improvement with tDCS applied over M1 while the other two failed to demonstrate significant effects compared to placebo.

**Conclusions:**

rTMS, applied to M1, DLPFC or S2, is a promising approach for the treatment of chronic orofacial pain. Moreover, tDCS targeting M1 seems to be also effective in chronic orofacial pain treatment. The included studies used a wide variety of therapeutic protocols. In addition, most of them used small sample sizes, with a high risk of biases in their methodologies, thus producing a low quality of evidence. The results indicate that further research should be carried out with caution and with better-standardized therapeutic protocols.

## Introduction

A considerable part of the general population will experience some episode of orofacial pain during their lifetime [1, 2], but only some of these will develop chronic orofacial pain. Orofacial pain is a highly generic term that covers a group of complex disorders, which comprise both nociceptive and neuropathic conditions such as temporomandibular disorders (TMD), trigeminal neuralgia (TN), postherpetic neuralgia (PHN), glossopharyngeal neuralgia, atypical facial pain (AFP), and burning mouth syndrome (BMS) [3]. According the International Association for the Study of Pain (IASP) classification of chronic pain for the International Classification of Diseases 11 (ICD-11), chronic primary headache or orofacial pain is characterized by “headache or orofacial pain that occurs on at least 15 days per month for longer than three months in the mouth and face area” [4]. In addition, patients are diagnosed according to the orofacial pain phenotype present within the last year [4]. The multifaceted pathophysiological mechanisms involved in the pathogenesis of this group of pathologies have not been yet elucidated. In addition, the coexistence of psychological factors, along with the vast number of peripheral and central mechanisms (e.g. functional and structural neuroplasticity, more specifically peripheral and central sensitization), make the treatment of orofacial pain extremely challenging [5, 6]. Therefore, the classification as well as the most appropriate therapies applied to treat orofacial pain and, in particular, chronic orofacial pain have been extensively reviewed and recently updated [4]. Epidemiological studies have explored the prevalence of chronic orofacial pain. For instance, in one of those studies, Aggarwal et al., 2019 [7] reported a prevalence as high as 7%.

Conservative treatments have been considered the first approach to treat both acute and chronic orofacial pain. Considering the heterogeneity of these conditions, the therapeutic protocol depends strongly on an accurate clinical diagnosis. For instance, even chronic TMD, is still a generic term including both myogenic and arthrogenic conditions, with completely different pathophysiological mechanisms. This heterogeneity must also be considered and fully understood when evaluating the efficacy of the therapies recommended for each disorder. Overall, robust scientific evidence suggests the use of noninvasive treatments such self-management interventions, presents high degree of efficacy to treat TMD [8]. The conservative treatments, included physical self-regulation, psychosocial (cognitive and behavioral] self-regulation and education, should be considered as the first-line treatment for primary chronic orofacial pain [8–10]. However, despite the great advances in TMD treatments, the effectiveness of more invasive treatments still cannot be determined by solid scientific evidence [11, 12]. The situation is similar with BMS. While it has been suggested that, capsaicin or topical clonazepam may be effective in the treatment of this neuropathic condition, the lack of more robust scientific evidence still does not support the use of these therapies in the treatment of BMS [11, 13]. Conversely, carbamazepine or oxcarbazepine are recommended as first-choice drugs to treat TN, with strong scientific evidence supporting their clinical use in this disease [14]. However, still according to the guidelines for TN developed by the European Academy of Neurology [14], specific surgical procedures may also be recommended in the treatment of this disease, when the related pain is not adequately controlled by clinical management or when the pharmacological approach is poorly tolerated by the affected patients.

In the early 1990s, Tsubokawa et al. [15, 16] introduced an alternative method to relieve chronic pain. This method, termed Motor Cortex Stimulation (MCS), uses intracranial electrodes to directly modulate the brain activity. Since then, MCS has been used in the treatment of chronic pain syndromes, including chronic orofacial pain disorders. Some studies suggest that it reduces pain more than other methods of brain stimulation. However, its efficacy still lacks scientific evidence [17]. In addition, MCS is a more invasive and expensive strategy, which limits its large-scale use. In contrast, Transcranial Magnetic Stimulation (TMS) and Transcranial Direct Current Stimulation (tDCS) are safe and low-cost approaches that, according to some studies, provide long-lasting pain relief [18, 19]. In brief, tDCS uses a constant low-amperage (usually 1–2 mA or even up to 3 mA) electric current that is delivered to the central nervous system through surface electrodes (e.g. anode or positive pole, and cathode or negative pole). These electrodes are positioned on the scalp [20]. The traditional technique (often referred to as conventional tDCS) uses larger electrodes (5x7 cm) to target different cortical regions, according to the disease to be treated. They all follow 10–10 or 10–20 electroencephalogram (EEG) landmarks. The most conventional setups applied are M1-SO (also referred to as motor cortex-supraorbital, with the anode electrode positioned over C3 and the cathode electrode positioned over Fp2); DLPFC (dorsolateral prefrontal cortices, with the anode positioned over F3 and the cathode over F4); and Cz-Oz (vertex-occipital cortex, with the anode placed over Cz and the cathode over Oz). More recently, a variation of the procedure, using smaller (ring) electrodes, has been developed and applied in some studies. The purpose of this variation of the original tDCS technique is to increase the focality and therefore redirect the majority of the electrical current to the motor cortex, which would be more anatomically related to the original target of the MCS technique originally published by Tsubokawa et al. [15, 16]. These methods have been collectively defined as High-Definition tDCS or HD-tDCS [21, 22]. On the other hand, TMS increases or decreases the neuronal activity by causing changes in magnetic fields [22]. Strong effects depolarize neurons, triggering action potentials. Low-intensity TMS can stimulate low-threshold inhibitory interneurons, whereas higher intensities excite projection neurons [23]. TMS Pulses may be applied singly; however, for therapeutic use, multiple pulses are applied rapidly (termed repetitive transcranial magnetic stimulation or rTMS) [24].

The precise antinociceptive mechanisms of tDCS and TMS are still not completely understood. It has been suggested that modulation of top-down circuits as well as the activation of diffuse brain regions might play a role [25]. These methods might modulate the cortical excitability, inducing the activation of endogenous pain-modulating systems as well as synaptic neuroplasticity [21, 25–28].

Several factors may interfere with the effectiveness of TMS or tDCS, including the type of intervention, cortical region targeted, and stimulation parameters (e.g. duration, intensity and frequency) [29–37]. These factors contribute to the contradictory findings of studies that analyzed the effectiveness of TMS and tDCS to treat chronic-pain syndromes [38, 39]. The clinical diagnosis is an additional feature that may directly affect the results, considering the enormous differences in the etiology, physiopathology, and clinical aspect of each chronic-pain syndrome.

The aims of this systematic review were to: (1) analyze the effectiveness of TMS and tDCS in the treatment of chronic orofacial pain, and (2) determine their most effective parameters. Therefore, the goal of the current study included the analysis of the following parameters: age, gender and number of participants, diagnostic criteria, pain, type of neuromodulatory technique (tDCS or TMS), targeted cortical region, frequency and number of sessions, intensity of stimulation, follow-up and washout periods (only for cross-over studies), and the characteristics of the placebo or therapy used for comparison.

## Materials and methods

### Study design and search strategy

This systematic review was registered in the PROSPERO database under the protocol CRD42018090774, and followed the recommendations of the PRISMA statement for systematic review reports [40]. This information is listed in S1 Checklist.

The search strategy was assisted by a librarian (DMTP), an expert in systematic reviews. The strategy combined terms found in the controlled vocabulary of MeSH (Medical Subject Headings) and terms representing synonyms with significant occurrences in major databases. The strategy was conducted according to the specifications of each database, where the Boolean operators “AND” and “OR” were used to combine keywords. The electronic searches were conducted in March 2018, using the following databases: MEDLINE (via PubMed), Scopus, Web of Science, Cochrane Library, EMBASE, LILACS, BBO (Brazilian Library of Dentistry), SIGLE (System for Information on Grey Literature in Europe) and CINAHL Database. All titles found in the search were imported into the EndNote Web reference manager to catalogue the references and automatically remove duplicate records. Multiple occurrences that remained were deleted manually. When documents recorded as conference abstracts were found, the authors of these documents were contacted to request full access to the study protocol, methods, and main findings of the study. The search strategies used are listed in Table 1. The manual search was performed in the reference list of articles included in this study.

**Table 1:** Database and search strategies.

**PubMed**	(Transcranial Magnetic Stimulation[mh] or Transcranial Magnetic Stimulation[tiab] or Magnetic Stimulation Transcranial[tiab] or TMS[tiab] or Transcranial Direct Current Stimulation[mh] or Transcranial Direct Current Stimulation[tiab] or tDCS[tiab] or Electrical Stimulation Transcranial[tiab]) AND (Facial Pain[mh] or Facial Pain[tiab] or Orofacial Pain[tiab] or Myofascial Pain[tiab] or Temporomandibular Joint[mh] or Temporomandibular Joint[tiab] or Temporomandibular Joint Disorders[mh] or TMJ[tiab] or TMJ Disorder*[tiab] or Jaw Diseases[mh] or Diseases Jaw[tiab] or Trigeminal Neuralgia[mh] or Trigeminal Neuralgia[tiab] or Trigeminal Nerve Diseases[mh] or Neuropathy Trigeminal[tiab] or Facial Neuralgia[mh] or Neuralgias Facial[tiab] or Neuralgia Postherpetic[mh] or Postherpetic Neuralgia[tiab] or Herpetic Neuralgia[Tiab] or Burning Mouth Syndrome[mh] or Mouth Syndromes Burning[tiab] or Neuritis[mh] or Neuritis[tiab] or Causalgia[mh] or Causalgia[tiab] or Deafferentation Pain[tiab])
**Web of Science**	("Facial Pain" or "Orofacial Pain" or "Myofascial Pain" or "Temporomandibular Disorders" or "Temporomandibular Joint" or TMJ or “Jaw Diseases" or "Trigeminal Neuralgia" or "Trigeminal Nerve Diseases" or "Trigeminal Neuropathy " or "Facial Neuralgia" or "Postherpetic Neuralgia " or "Herpetic Neuralgia" or "Mouth Syndromes Burning" or Neuritis or Causalgia or "Deafferentation Pain") AND (“Transcranial Magnetic Stimulation” or “Magnetic Stimulation Transcranial” or TMS or “Transcranial Direct Current Stimulation” or tDCS or “Electrical Stimulation Transcranial”)
**Scopus**	("Facial Pain" or "Orofacial Pain" or "Myofascial Pain" or "Temporomandibular Joint" or tmj or "Jaw Diseases" or "Trigeminal Neuralgia" or "Trigeminal Nerve Diseases" or "Trigeminal Neuropathy" or "Facial Neuralgias" or "Postherpetic Neuralgia" or "Herpetic Neuralgia" or "Burning Mouth" or neuritis or causalgia or "Deafferentation Pain") AND ("Transcranial Magnetic Stimulation" or "Magnetic Stimulation Transcranial" or tms or "Transcranial Direct Current Stimulation" or tDCS or "Electrical Stimulation Transcranial")
**Cochrane**	#1 MeSH descriptor: [Transcranial Magnetic Stimulation] explode all trees or "Transcranial Magnetic Stimulation" or TMS or MeSH descriptor: [Transcranial Direct Current Stimulation] explode all trees or Transcranial Direct Current Stimulation" or tDCS. #2 MeSH descriptor: [Facial Pain] explode all trees or "Facial Pain" or "Orofacial Pain" or "Myofascial Pain" or MeSH descriptor: [Temporomandibular Joint] explode all trees or "Temporomandibular Joint" or TMJ or MeSH descriptor: [Temporomandibular Joint Disorders] explode all trees or "Temporomandibular Joint Disorders" or "TMJ Disorders" or MeSH descriptor: [Jaw Diseases] explode all trees or "Jaw Diseases" or MeSH descriptor: [Trigeminal Neuralgia] explode all trees or "Trigeminal Neuralgia" or MeSH descriptor: [Trigeminal Nerve Diseases] explode all trees or "Trigeminal Nerve Diseases" or "Trigeminal Neuropathy" or MeSH descriptor: [Facial Neuralgia] explode all trees or "Facial Neuralgia" or MeSH descriptor: [Neuralgia, Postherpetic] explode all trees or "Postherpetic Neuralgia" or "Herpetic Neuralgia" or MeSH descriptor: [Burning Mouth Syndrome] explode all trees or "Burning Mouth Syndrome" or MeSH descriptor: [Neuritis] explode all trees or Neuritis or MeSH descriptor: [Causalgia] explode all trees or Causalgia or "Deafferentation Pain". #1 AND #2
**LILACS**	(tw:((mh:("Transcranial Magnetic Stimulation")) or (tw:("Transcranial Magnetic Stimulation")) or (tw:("Estimulação Magnética Transcraniana")) or (tw:(TMS)) or (mh:("Transcranial Direct Current Stimulation")) or (tw:("Transcranial Direct Current Stimulation")) or (tw:("Estimulação Transcraniana por Corrente Contínua")) or (tw:(tDCS)) or (tw:(ETCC)))) AND (tw:((mh:("Facial Pain")) or (tw:("Facial Pain")) or (tw:("Dor Facial")) or (tw:("Orofacial Pain")) or (tw:("Dor Orofacial")) or (tw:("Myofascial Pain")) or (tw:("Dor Miofascial")) or (mh:("Temporomandibular Joint")) or (tw:("Temporomandibular Joint")) or (tw:("Articulação Temporomandibular")) or (tw:(TMJ)) or (tw:(ATM)) or (mh:("Temporomandibular Joint Disorders")) or (tw:("TMJ Disorders")) OR (tw:("Transtornos da ATM")) or (mh:("Jaw Diseases")) or (tw:("Doenças Maxilomandibulares")) or (tw:("Diseases Jaw")) or (mh:("Trigeminal Neuralgia")) or (tw:("Trigeminal Neuralgia")) or (tw:("Neuralgia do Trigêmeo")) or (mh:("Trigeminal Nerve Diseases")) or (tw:("Neuropathy Trigeminal")) or (tw:("Doenças do Nervo Trigêmeo")) OR (mh:("Facial Neuralgia")) or (tw:("Neuralgias Facial")) or (tw:("Neuralgia Facial")) or (mh:("Neuralgia, Postherpetic")) or (tw:("Postherpetic Neuralgia")) or (tw:("Herpetic Neuralgia")) or (mh:("Burning Mouth Syndrome")) or (tw:("Mouth Syndromes Burning")) OR (tw:("Síndrome da Ardência Bucal")) or (mh:(Neuritis)) or (tw:(Neuritis)) or (mh:(Causalgia)) or (tw:(Causalgia)) or (tw:("Deafferentation Pain"))))
**BBO**	(tw:((mh:("Transcranial Magnetic Stimulation")) or (tw:("Transcranial Magnetic Stimulation")) or (tw:("Estimulação Magnética Transcraniana")) or (tw:(TMS)) or (mh:("Transcranial Direct Current Stimulation")) or (tw:("Transcranial Direct Current Stimulation")) or (tw:("Estimulação Transcraniana por Corrente Contínua")) or (tw:(tDCS)) or (tw:(ETCC)))) AND (tw:((mh:("Facial Pain")) or (tw:("Facial Pain")) or (tw:("Dor Facial")) OR (tw:("Orofacial Pain")) or (tw:("Dor Orofacial")) or (tw:("Myofascial Pain")) or (tw:("Dor Miofascial")) or (mh:("Temporomandibular Joint")) or (tw:("Temporomandibular Joint")) or (tw:("Articulação Temporomandibular")) or (tw:(TMJ)) or (tw:(ATM)) or (mh:("Temporomandibular Joint Disorders")) or (tw:("TMJ Disorders")) or (tw:("Transtornos da ATM")) or (mh:("Jaw Diseases")) or (tw:("Doenças Maxilomandibulares")) or (tw:("Diseases Jaw")) or (mh:("Trigeminal Neuralgia")) or (tw:("Trigeminal Neuralgia")) OR (tw:("Neuralgia do Trigêmeo")) or (mh:("Trigeminal Nerve Diseases")) or (tw:("Neuropathy Trigeminal")) or (tw:("Doenças do Nervo Trigêmeo")) or (mh:("Facial Neuralgia")) or (tw:("Neuralgias Facial")) or (tw:("Neuralgia Facial")) or (mh:("Neuralgia, Postherpetic")) or (tw:("Postherpetic Neuralgia")) or (tw:("Herpetic Neuralgia")) or (mh:("Burning Mouth Syndrome")) or (tw:("Mouth Syndromes Burning")) or (tw:("Síndrome da Ardência Bucal")) or (mh:(Neuritis)) or (tw:(Neuritis)) or (mh:(Causalgia)) or (tw:(Causalgia)) or (tw:("Deafferentation Pain"))))
**EMBASE**	('transcranial magnetic stimulation'/exp OR 'transcranial magnetic stimulation' OR 'transcranial magnetic stimulation':ab,ti OR 'tms':ab,ti OR 'transcranial direct current stimulation'/exp OR 'transcranial direct current stimulation' OR 'transcranial direct current stimulation':ab,ti OR 'tdcs':ab,ti) AND ('causalgia'/exp OR 'causalgia' OR 'causalgia':ab,ti OR 'deafferentation pain':ab,ti OR 'neuritis'/exp OR 'neuritis' OR 'neuritis':ab,ti OR 'burning mouth syndrome'/exp OR 'burning mouth syndrome' OR 'burning mouth syndrome':ab,ti OR 'postherpetic neuralgia'/exp OR 'postherpetic neuralgia' OR 'postherpetic neuralgia':ab,ti OR 'herpetic neuralgia':ab,ti OR 'facial neuralgia'/exp OR 'facial neuralgia' OR 'facial neuralgia':ab,ti OR 'trigeminal nerve disease'/exp OR 'trigeminal nerve disease' OR 'neuropathy trigeminal':ab,ti OR 'trigeminal neuralgia'/exp OR 'trigeminal neuralgia' OR 'trigeminal neuralgia':ab,ti OR 'jaw disease'/exp OR 'jaw disease' OR 'temporomandibular joint disorder'/exp OR 'temporomandibular joint disorder' OR 'tmj disorders':ab,ti OR 'temporomandibular joint'/exp OR 'temporomandibular joint' OR 'temporomandibular joint':ab,ti OR 'tmj':ab,ti OR 'face pain'/exp OR 'face pain' OR 'facial pain':ab,ti OR 'orofacial pain':ab,ti OR 'myofascial pain':ab,ti)
**Cinahl**	#1 S1 MH Transcranial Magnetic Stimulation or TI Transcranial Magnetic Stimulation or AB Transcranial Magnetic Stimulation or TI Magnetic Stimulation Transcranial or AB Magnetic Stimulation Transcranial or MH Transcranial Direct Current Stimulation or TI Transcranial Direct Current Stimulation or AB Transcranial Direct Current Stimulation or TI tDCS or AB tDCS. #2 MH Facial Pain or TI Facial Pain or AB Facial Pain or TI Orofacial Pain or AB Orofacial Pain or TI Myofascial Pain or AB Myofascial Pain or MH Temporomandibular Joint or TI Temporomandibular Joint or AB Temporomandibular Joint or MH Temporomandibular Joint Diseases or TI Temporomandibular joint disorder* or AB Temporomandibular joint disorder* or TI TMJ or AB TMJ or MH Jaw Diseases or MH Trigeminal Neuralgia or TI Trigeminal Neuralgia or AB Trigeminal Neuralgia or MH Trigeminal Nerve Diseases or TI Neuropathy Trigeminal or AB Neuropathy Trigeminal or MH Facial Neuralgia or TI Neuralgia Postherpetic or AB Neuralgia Postherpetic TI Postherpetic Neuralgia or AB Postherpetic Neuralgia or TI Herpetic Neuralgia or AB Herpetic Neuralgia or MH Burning Mouth Syndrome or TI Burning Mouth Syndrome* or AB Burning Mouth Syndrome* or MH Neuritis or TI Neuritis or AB Neuritis or MH Causalgia. #1 AND #2

#### Article selection

Titles and abstracts found in the search were evaluated independently by two researchers (NRF and MFD), and any disagreement was resolved by consensus. Relevant articles were read in full and only those that met the inclusion criteria were selected. Selected articles were evaluated and possible disagreements discussed; if any disagreement persisted, a third researcher (YNJ) was consulted. The inter-individual kappa was calculated after removal of duplicates, using the software SPSS 20.0 (SPSS Inc., Chicago, USA).

#### Inclusion criteria

The following eligibility criteria were applied, based on the PICOS strategy [30]:

■ Population (P): Adults over 18 years old, with a clinical diagnosis of orofacial pain of neuropathic or nociceptive origin. Only studies that adopted a clear diagnostic criterion such as the Research Diagnostic Criteria for Temporomandibular Disorders (RDC-TMD), the Diagnostic Criteria for TMD (DC/TMD), or the International Classification of Headache Disorders (ICHD) were included.
■ Intervention (I): Transcranial Magnetic Stimulation (TMS) and/or transcranial Direct Current Stimulation (tDCS). Any stimulation protocol, regardless of the number of sessions, stimulus intensity, duration, frequency, and anatomical location, was considered in the inclusion criteria.
■ Comparison (C): Placebo or any therapy used in orofacial pain management.
■ Outcome (O):
  ○ Primary outcomes:
    – Pain or discomfort evaluated through a validated scale;
    – Functional limitation;
  ○ Secondary outcomes:
    – Quality of life;
    – Tolerance to treatment;
    – Somatosensory or electrophysiological changes;
  ○ Adverse effects–Any unpleasant effect, during or after therapy, was included.
■ Study design (S): Randomized clinical trial.

#### Assessment of bias risk

Each article was assessed for the risk of bias, using the Collaboration's 'Risk of bias' tool [41] described in the Cochrane Handbook for Systematic Reviews of Interventions, version 5.1. This tool includes five bias domains: selection, performance, detection, attrition, and publication. Several methodological parameters of each study were evaluated to determine the risk of bias. The parameters analyzed were: random sequence generation/ concealed allocation (selection bias), blinding of participants and personnel (performance bias), blinding of outcome assessment (detection bias), incomplete outcome data addressed (attrition bias), and selective reporting (publication bias). The risk of bias for each article was judged as “low” or “high” risk of bias. In addition, “unclear” was used to indicate either a lack of information or uncertainty regarding the potential risk of bias. During this phase, each study was evaluated by two independent researchers (NRF and MFD) and the results were compared. Again, in case of disagreement, a third researcher (YNJ) was consulted and a consensus decision was made. In an attempt to resolve the “unclear” domains, in cases of missing data, the authors were contacted two times by electronic message and requested to provide additional information. If the authors answered the questions, the domains were judged as “low” or “high” risk of bias; and if the authors did not reply, the domain remained as “unclear”.

The strength of the evidence of the included studies was assessed using the Grading of Recommendations Assessment, Development and Evaluation (GRADE) tool [42].

#### Obtaining the data

The original data of the included articles were summarized on data collection forms, which were specifically designed to address the questions of this systematic review. The development of data collection forms aims to include all relevant information for a systematic review. In order to determine the effectiveness of the data collection forms, a pilot test was conducted using a representative sample of the studies. This test was important to identify any missing data not included in the forms, or any irrelevant data. The design and pilot testing of the data collection forms were performed according to the Cochrane Handbook for Systematic Reviews of Interventions [40]. The following parameters were analyzed: age, gender and number of participants, diagnostic criteria, pain, type of neuromodulatory technique applied, targeted cortical area, frequency and number of sessions, intensity of stimulation, follow-up and washout periods (for cross-over studies), and the characteristics of the placebo or therapy used for comparison. In cases of missing relevant data (e.g. rates of pain reduction found in each study group), the contact author of the referred study was reached twice by email.

## Results

### Studies included

**Fig 1:**
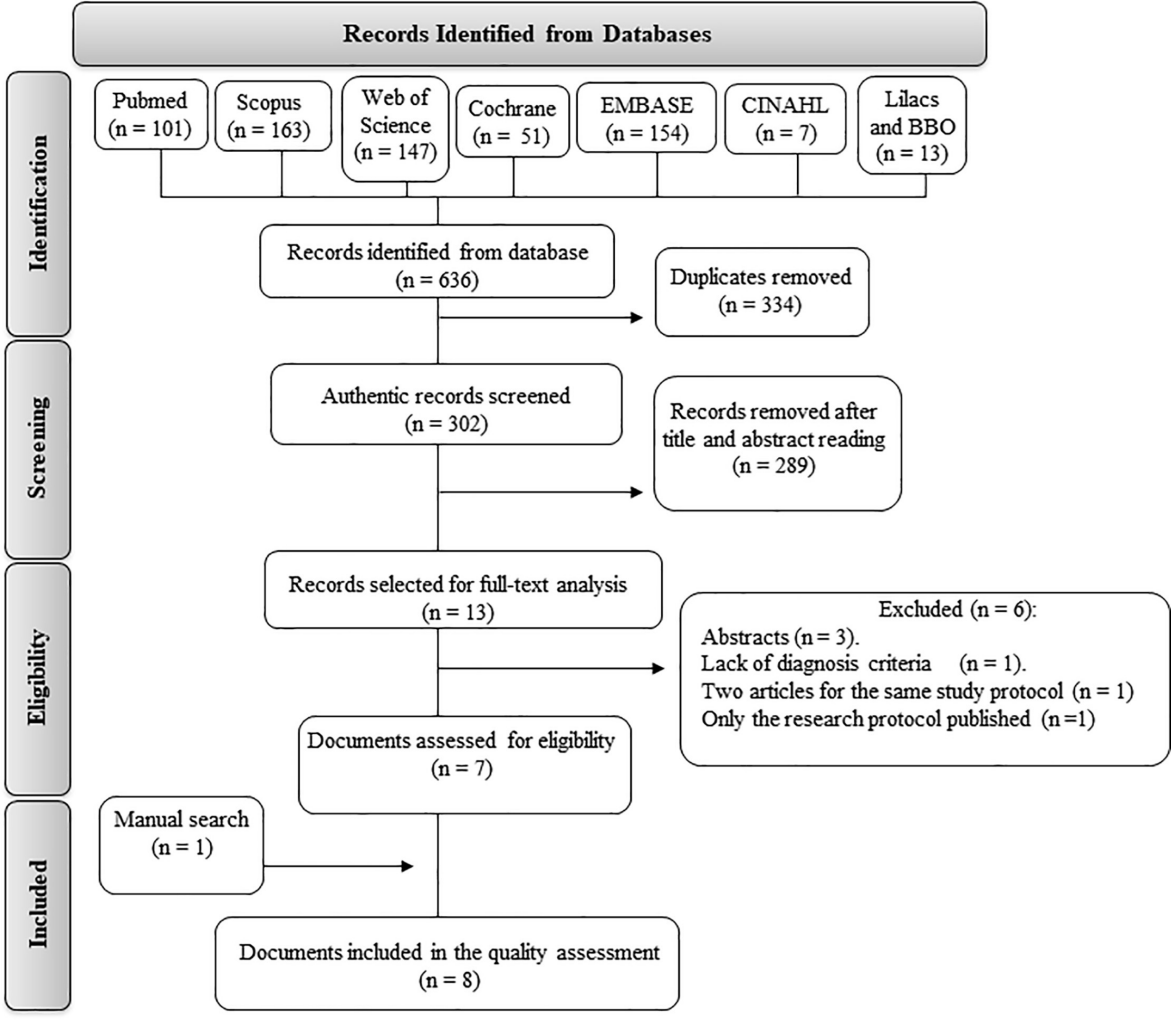
Flow diagram of the search strategy, with the number of studies selected in each database and in each phase of this systematic review.

The search strategy resulted in 636 records. Following the removal of duplicates (334), 302 documents remained. Analysis of the title and abstract resulted in the exclusion of 289 documents. The 13 remaining documents were read in full. The kappa of agreement was 0.75. Three of these documents were conference abstract: Lindholm et al., 2013, Jaeaeskelaeinen et al., 2014 and Obermann et al., 2014 [43–45], all relating to articles included in this review, and were excluded from the analysis. Another article, Fricova et al., 2013, was excluded due to the lack of a diagnosis criteria, [46]. The articles published by Lindholm et al., 2015 [34]and Lindholm et al., 2016 [33] represented the same clinical study, and therefore, although they evaluated different variables, were considered as a single clinical trial. Brandão Filho et al., 2015 [29] published two documents representing the same clinical trial. One of these contained only the study protocol [47] and was published as a research article, while the results of the study were found only in a PhD thesis, which was recovered through a manual search. A total of 10 documents (representing 8 RCTs) were included in this systematic review. A flow diagram of the search strategy is presented in Fig 1. A total of 219 participants were enrolled in the studies that were included. Of these patients, 75 were diagnosed with myofascial TMD and 133 were diagnosed with neuropathic orofacial pain. To simplify the analysis, the studies were divided based on the therapeutic method used to treat the orofacial-pain disorder evaluated. The demographic characteristics of the patients included in the studies, as well as the duration of the orofacial pain, the use of previous therapies prior to tDCS or TMS, and the inclusion criteria of each RCT are described in Table 2. No additional information that could have been used to perform a quantitative analysis was obtained after reaching the contact authors of the included studies by email. Therefore, a meta-analysis was not conducted.

**Table 2:** Main characteristics of the sample in each included study.

Study	Inclusion criteria	Clinical characteristics	Previous treatments	Pain duration
Khedr et al., 2005 [35]	“Unilateral chronic neuropathic pain. The diagnosis was based on the criteria of the International Association for the Study of Pain (IASP).”	16 women and 8 men (mean (SD) age 51.5 (10.7) years).	”All patients had been treated with various medications, including anticonvulsants, narcotic or non-narcotic analgesics and antidepressants, without satisfactory pain control. Three of the patients with TN had persistent pain even after microvascular decompression”.	Mean duration of illness of 39 months.
Lindholm et al., 2015 [34] and Lindholm et al., 2016 [33]	“Chronic daily neuropathic pain ≥4 in severity using a numeric rating scale (NRS); Diagnosis was based on International Criteria for Headache Disorders (ICDH 2013).”	N = 16, two of them male, with a mean age of 59 (range, 37–74).	“All patients had been diagnosed and treated for neuropathic orofacial pain in Turku University Hospital. All patients were suffering from severe chronic drug-resistant pain.”	Mean age of 10,44 years (not provided in the article).
Galhardoni et al., 2014 [30]	“Patients diagnosed with Atypical Facial Pain under treatment for at least three months. Persistent pain after at least two years of pharmacological treatment without adequate control. Pain higher than 6 at the VAS for at least three months.”	N = 19, females and males, with a mean age of 53,28±12,39 (28–78 years).	“All patients had received at least two pharmacological treatments. 44,8% had at least one concomitant treatment.”	Mean age of 13,5 ±11,04.
Umezaki et al., 2015 [48]	“BMS diagnosis; patients experienced daily and deep bilateral burning sensation of the oral mucosa, burning sensation for at least 4–6 months…”	All subjects were women except for one man in both the real and sham groups. Total mean age was 63.9 years.	“Selective serotonin reuptake inhibitors (SSRIs) were prescribed to around 40% of the patients, but these did not adequately relieve the BMS pain.”	Mean duration of illness was 63.4 months.
Brandão Filho et al., 2015 [29]	“…had a diagnosis of muscular TMD pain according to IA and IB, Axis I of the Research Diagnosis Criteria for Temporomandibular Disorders; had a VAS pain score of four or greater, presented pain regularly for six months or longer; had a State-Trait Anxiety Inventory (STAI) score of more than 42.”	All subjects were women, mean age was 36,5 years (± 12,3).	None of the subjects was under pharmacological treatments during the study. No reports regarding previous therapies.	Mean duration of pain was 7,1 (± 4,9)
Donnell et al., 2015 [31]	“Daily chronic TMD pain and dysfunction for at least one year matching RDC/TMD Axis I Group I: Myofascial pain diagnosis not adequately controlled by previous conventional therapies (TMJ open-surgery naïve) for more than 1 year; with self-reported pain score of at least 3 on VAS …”	All subjects were women. Mean age was 35,2 years.	No reports regarding conservative therapies adopted.	Pain Duration: higher than one year.
Hagenacker et al., 2014 [32]	“Classical TN with or without concomitant permanent pain according to the beta-version of the 3rd edition of the International Classification of Headache Disorders (ICHD-3).”	5 men and 4 women. Mean age of 63 years (range: 49–82 years).	“All patients had additional medication with different combinations of antiepileptic drugs. None of the investigated patients had an invasive procedure prior to study inclusion.”	Mean duration of illness was 9.78 years (2 to 27 years).
Oliveira et al., 2015 [37]	“TMD diagnosis based on RDC/TMD Ia or Ib. All patients had to present mean orofacial mean pain intensity equal to or over 4/ 10 on a VAS, during the last 6 months.”	3 men and 29 women. Mean age of 23,80± 7,30 (active group) and 25,50 ± 6,30 (sham group)	No reports regarding previous therapies. Volunteers that had received previous treatment of physical therapy could not be included in the study.	Mean duration of illness was 29,80 ± 17,10 months (active group) and 33,70 ± 22,80 months (sham group).

#### Transcranial direct current stimulation

Donnell et al., 2015 [31], Oliveira et al., 2015 [37], Brandão Filho et al., 2015 [29] and Hagenacker et al., 2014 [32] applied tDCS to treat orofacial pain. Three of these evaluated the effects of tDCS on myofascial TMD. Only the study by Hagenacker et al., 2014 [32] evaluated the effects of tDCS on patients diagnosed with neuropathic orofacial pain, more specifically classic trigeminal neuralgia. The research protocols varied widely among the studies, with important differences in the number of sessions, anatomical site of stimulation, type of electrodes, setup, and intensity of the electrical current delivered. The results are summarized in Table 3.

**Table 3:** Main findings of the tDCS studies.

Reference	Diagnosis	N	Stimulation	Site	Arrangement	Results
Hagenacker et al., 2014 [32]	TN	10	Anodal-tDCS	M1	Cross-over 1 mA and sham 20 min/session 14 sessions	Pain (VRS) difference comparing anodal vs sham-tDCS was 29% (p = 0.008): VRS decreased after anodal stimulation compared to baseline by 18%, while sham stimulation led to an 11% increase of VRS. Attack frequency was not significantly decreased between sham or anodal stimulation. No comparison between baseline and end of treatment was reported. Attack frequency decreased in both groups but not significantly.
Donnell et al., 2015 [31]	TMD	24	HD-tDCS (2x2) Active (n = 12) or sham (n = 12)	M1	Parallel-group 2 mA or sham 20 min/session 5 sessions	Pain (VAS) decreased over 50% in 75% of the active group and in 33,3% of the control group. No changes in emotional outcomes were found between groups. There were significant differences between placebo and HD-tDCS were found for: responders presenting pain relief higher than 50% in the VAS at the four-week of the follow-up; pain-free mouth opening at one-week follow-up and sectional pain area, intensity and their sum contralateral to stimulated M1 during the period of treatment.
Oliveira et al., 2015 [37]	TMD	32	Anodal-tDCS + physical therapy Active (n = 16) or sham (n = 16)	M1	Parallel-group 2 mA or sham 20 min/session 5 sessions	Pain (VAS) and Quality of life improved in both groups. Nevertheless, with no significant difference. In the last session, 75% of the active and 37,5% of the control group were TMD symptom free and no longer classified as having a diagnosis of TMD (RCD/TMD). After 5 months: both groups maintained with lower pain levels; 29% of the active and 50% of the control group continued to perform the exercises.
Brandão Filho et al., 2015 [29]	TMD	15	Cathodal-tDCS	DLPFC	Cross-over 1 mA, 2 mA and sham 20 min/session 1 sessions	Pain (VAS) was not significantly decrease after any stimulation. Anxiety has decreased significantly in all types of stimulation. Nonetheless, with no difference between them.

Several variables were analyzed in the studies that explored the efficacy of tDCS to treat orofacial pain. Nevertheless, in most of those studies, a significant difference between active and sham groups could not be demonstrated. Only pain, when assessed by a tool called PainTrek and the degree of mouth opening, both variables investigated in the study published by Donnell et al., 2015 [31], showed a significant improvement in the active group, when compared to the placebo group. The same study also evaluated pain through the short form of the McGill Pain Questionnaire (SF-MPQ); mood changes, through the Positive and Negative Affect Schedule (PANAS); as well as changes in the mandibular kinematics. None of these variables were significantly different between groups (Active x placebo tDCS). Oliveira et al., 2015 [37] investigated the effects of tDCS on pressure pain thresholds (PPT) of the temporomandibular joint (TMJ) and cervical muscles. No group differences were found. Brandão Filho et al., 2015 [29] also explored the effects of tDCS on PPT and on other Quantitative Sensory Testing parameters (e.g. mechanical detection and pain thresholds, using Von Frey filaments). Again, they detected no significant differences among the different groups (sham, 1 mA and 2 mA tDCS). In the study by Hagenacker et al., 2014 [32], the frequency of attacks and the impact of the therapy on the treatment of trigeminal neuropathic pain was investigated through pain-related evoked potentials (PREP) and the nociceptive blink reflex (nBR). No significant group differences were detected.

The longest follow-up period was five months, in the study by Oliveira et al., 2015 [37]. However, that follow-up was conducted by phone and the researchers were able to contact only 87% of the subjects in the active tDCS group and 62% of the subjects in the sham group. In addition, the only variable assessed at that time was pain, since the other variables could be evaluated only during the treatment period. Donnell et al., 2015 [31] conducted two follow-ups. The first follow-up was performed one week after the end of the treatment and the second follow-up four weeks after the end of the treatment. Hagenacker et al., 2014 [32] performed a single follow-up, three days after the end of the treatment. Brandão Filho et al. 2015 [29] evaluated the subjects only within the treatment period.

tDCS was considered well tolerated, without producing significant adverse effects. Donnell et al., 2015 [31] reported that the most frequent adverse events found in their study were headache, neck pain, burning-scalp sensation, scalp pain, tingling, skin redness, sleepiness, difficulty in concentrating, and mood change. No significant difference was found between the two groups (active and sham) regarding the presence of these side effects. On the other hand, Oliveira et al., 2015 [37] reported the occurrence of skin burns in one subject, due to acne in the supraorbital region. Brandão Filho et al., 2015 [29] did not observe significant adverse effects. In the two types of active intervention used in this study (1mA and 2mA), the most common was scalp redness. The authors found no statistically significant difference between the sham and active tDCS. Hagenacker et al., 2014 [32] reported that all patients tolerated tDCS, with no occurrence of adverse effects. The adverse effects reported in each tDCS /TMS article are summarized in S1 Table.

Donnell et al., 2015 [31] and Hagenacker et al., 2014 [32] did not assess the level of blinding achieved in their study. Oliveira et al., 2015 [37] requested the participants in their study to identify the treatment group to which they belonged. According to their results, 15 participants in the active tDCS group and seven in the sham tDCS group correctly guessed the type of treatment they were assigned. Brandão Filho et al., 2015 [29] assessed the blinding effectiveness using the Kappa level of agreement. Cohen's kappa coefficient (κ) is a measures inter-rater agreement for categorical items. It is considered a more robust measure than percent agreement calculation [29]. Overall, the guess rate for the type of intervention was considered low. The guess rate was 13.0% with 2mA tDCS, 11.1% with sham tDCS, and 20.4% with 1mA tDCS. The Kappa level of agreement also showed low agreement (r = 0.167; p = 0.10).

#### Transcranial magnetic stimulation

Galhardoni et al., 2014 [30]; Khedr et al., 2005 [35]; Lindholm et al., 2015 [34] and Umezaki et al., 2015 [48] evaluated the efficacy of repetitive TMS (rTMS) in the treatment of neuropathic orofacial pain, including BMS, AFP and TN. The stimulation protocols differed in the number of sessions, site of the stimulation, and the intensity and number of pulses per session. Table 4 summarizes the protocols and main findings of each TMS study included in this systematic review.

**Table 4:** Main findings of the TMS studies.

Reference	Sample	N	Stimulation	Site	Arrangement	Results
Khedr et al., 2005 [35]	TN PSP	24 25	rTMS Active (n = 14) or sham (n = 10) Figure-of-8 coil Painful side	M1	Parallel-group 20 Hz, 80% RMT 2000 pulses/session, Trains of 10s: 200 pulses /1min intertrain interval 5 sessions	Pain (VAS) reduced by 45% in the active group at the 5th session and maintained by 40% after 15 days of treatment. On the other hand, sham group declined 5% and 2%, respectively. There was a significant treatment x time interaction suggesting that real and sham rTMS promoted different effects on the VAS and LANSS scales. This difference remained for two weeks after the end of the treatment.
Galhardoni et al., 2014 [30]	AFP	33	rTMS Active (n = 15) or sham (n = 14) Figure-of-8 coils contralateral	M1	Parallel-group 10 Hz, 80% RMT 3000 pulses/session Trains of 20s: 100 pulses/ 20s intertrain interval. 13 sessions	Pain (VAS) and Quality of Life improved. Nevertheless, without significant difference between active and control groups.
Lindholm et al., 2015 [34] and Lindholm et al., 2016 [33]	TN AFP BMS	7 4 5	rTMS Active or sham Figure-of-8 coils S2: right side M1/S1: contralateral	M1/S1 (1) or S2 (2)	Cross-over 10 Hz, 90% RMT 1000 pulses/session Trains: 50 pulses/ 10s intertrain interval 3 sessions	Pain intensity (NRS) was lowest on the third day in the S2 TMS group. In addition, it was significantly lower in the S2 group compared to S1/M1 or sham groups. Quality of life showed a mild improvement. However, without significant difference. TMS had no effect on sleep, mood or depressive symptoms.
Umezaki et al., 2015 [48]	BMS	26	rTMS Active (n = 14) or sham (n = 12) Figure-of-8 coils Left side	DLPFC	Parallel-group 10 Hz, 110% RMT 30000 pulses/session Trains of 5s:10s intertrain interval 10 sessions (15 min)	Pain (VAS and SF-MPQ) were significantly different when comparing active and sham groups on the days 15 and 6. The results of PHQ9 suggested an overall improvement in both groups, without difference between groups.

Besides pain, Lindholm et al., 2015 [34] 2016 [33] studied the effects of rTMS on quality of life as well as the influence of functional polymorphisms on these effects. In the study by Lindholm et al., 2015 [34]; 2016 [33], none of the variables investigated were significantly different, when comparing the study groups. Galhardoni et al., 2014 [30] evaluated the quality of life. In agreement with the results of Lindholm et al., 2015 [34]; 2016 [33], no significant group differences were found. Conversely, Khedr et al., 2005 [35] only explored the effects of rTMS on pain. Overall, the TMS treatment was well tolerated in all studies. When present, side effects were mild and self-limited.

Among the rTMS studies included, the longest evaluation periods were used by Galhardoni et al., 2014 [30] and Umezaki et al., 2015 [48]. Galhardoni et al., 2014 [30] evaluated the subjects of their study for up to 60 days, counting from the start of the treatment. Umezaki et al., 2015 [48] performed three follow-ups (at days 15, 30 and 60 from the start of the treatment). Lindholm et al., 2015 [34] performed the last evaluation one month after the end of the treatment, while Khedr et al., 2005 [35] conducted only one follow-up, two weeks after the last treatment session.

Regarding adverse effects related to rTMS treatment, Lindholm et al., 2015 [34] observed discomfort associated with the contraction of the temporalis muscle in two participants in the active group. Umezaki et al., 2015 [48] reported the occurrence of headache at the beginning of treatment, which disappeared within two days in seven participants in the active group and in five participants in the sham group. Khedr et al., 2005 [35] reported an absence of adverse effects, while Galhardoni et al., 2014 [30] did not describe any evaluation of side effects in their study. The adverse effects reported in each tDCS /TMS article are summarized in S1 Table.

Blinding assessments were conducted only by Umezaki et al., 2015 [48] and Lindholm et al., 2015 [34]. Umezaki et al., 2015 [48] found that 10 of the 12 (83%) patients in the active rTMS group and four of the eight (50%) patients in the sham group guessed that they had been allocated to the active group. However, there were no significant differences between the two groups regarding the belief of group allocation. Lindholm et al., 2015 [34] found that six of the 16 participants correctly guessed the placebo stimulation, two because of muscle contraction caused by active rTMS, and four because of the beneficial effects produced by the active TMS treatment.

#### Risk of bias and quality of evidence

**Fig 2:**
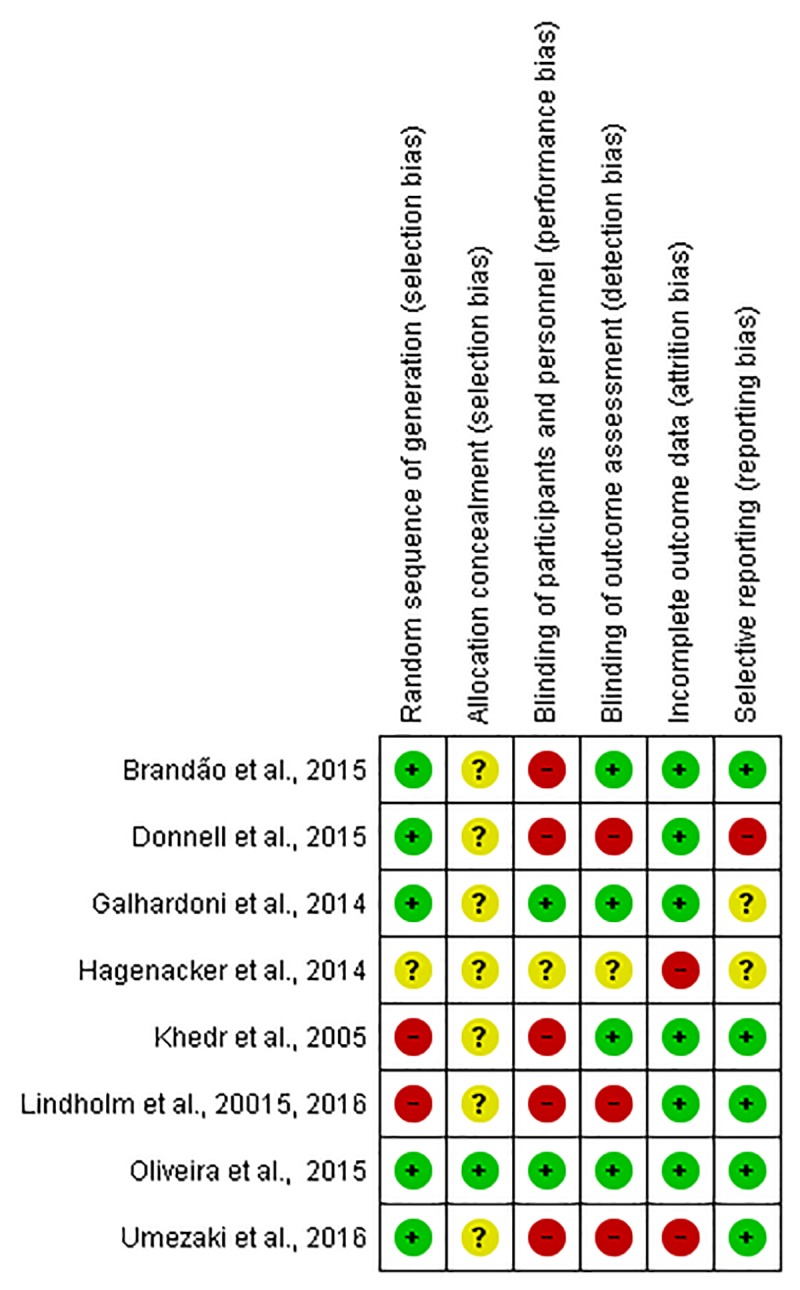
Risk-of-bias assessment. Red represents high risk of bias, yellow unclear risk, and green low risk in each domain evaluated.

Only the study by Oliveira et al., 2015 [28] showed a low risk of bias in all domains evaluated. All other studies showed a high or uncertain risk in at least two parameters of the risk-of-bias assessment. Fig 2 illustrates the risk-of-bias assessment in each domain of the studies included in this systematic review.

Regarding the strength of evidence overall classification, the GRADE tool demonstrated a low quality of evidence for both tDCS and rTMS studies (Table 5).

**Table 5:** Quality of evidence of non-invasive neuromodulation for the treatment of orofacial pain.

Certainty assessment							Summary of findings
№ of participants (studies)	Risk of bias	Inconsistency	Indirectness	Imprecision	Publication bias	Overall certainty of evidence	Summary of findings
tDCS (4 RCTs) Active group N = 53 Sham group N = 53	serious ^a^	serious ^b^	not serious	serious ^c^	all plausible residual confounding would suggest spurious effect, while no effect was observed	⨁⨁◯◯ LOW	Anodal M1-tDCS was effective in orofacial pain relief
rTMS (5 RCTs) Active group N = 59 Sham group N = 52	serious ^a^	serious ^b^	not serious	serious ^c^	all plausible residual confounding would suggest spurious effect, while no effect was observed	⨁⨁◯◯ LOW	rTMS is a promising approach for the treatment of orofacial pain, regardless of the cortical site of stimulation.

^a^ The respective item received a downgrade because the majority of the studies did not blind participants and personnel and due to the outcome assessing.

^b^ The item received a downgrade due to a large heterogeneity among the studies.

^c^ The item imprecision received a downgrade due to the major studies did not perform sample size calculation and the participant did not present the same level of orofacial pain in the baseline.

⨁⨁◯◯ represents the classification obtained in the evaluated categories (risk of bias, inconsistency, indirectness, imprecision).

## Discussion

The use of tDCS and TMS brain stimulation to treat acute and chronic pain conditions has increased significantly in recent years, followed by rapid growth in related studies. Nonetheless, some aspects of these therapies remain largely unknown, including their mechanisms of action as well as their clinical efficacy. The present systematic review included eight studies. Four of them evaluated the efficacy of rTMS and the other four assessed the efficacy of tDCS in treating orofacial-pain conditions. A total of 219 patients participated in these clinical trials; 75 participants had diagnoses of myofascial TMD and 133 had diagnoses of neuropathic orofacial pain. It was not possible to conduct a meta-analysis due to the wide variety of methods used in these studies. In cases of unmatched methodologies and impossible comparison between different applied treatments, a meta-analysis is not recommended. Therefore, the findings presented here are descriptive and qualitative. In addition, no additional information that could have been used to perform a quantitative analysis (in cases of missing data such as the rates of pain reduction found in each study group) was obtained after reaching the contact authors of the included studies by email. The tDCS studies varied in the intensity of the electrical current, site of stimulation and number of sessions applied. The rTMS studies varied not only in the number of sessions and the cortical region stimulated, but also in the frequency of pulses delivered.

Only two of the eight studies analyzed the efficacy of M1-rTMS compared to a placebo, with contradictory results. While Khedr et al., 2005 [35] demonstrated a significant improvement of pain with active TMS compared to a placebo, Galhardoni et al., 2014 [30] reported no significant difference between placebo and active treatments in any of the variables evaluated. Such divergences might be related to the use of different protocols/methodological designs and/or to the inclusion of different pain disorders. For instance, the two conditions evaluated, trigeminal neuralgia [35] and atypical facial pain [30], have different etiologies, pathophysiologies and clinical manifestations. Another highly important aspect is the difference between the clinical protocols of rTMS application. Khedr et al., 2005 [35] used a pulse rate well above that used by Galhardoni et al., 2014 [30], who in turn used more pulses per session and a larger number of sessions. In addition, some methodological limitations may have affected the results. Both studies were classified as having a high risk of bias in their risk-of-bias assessment (Fig 2).

Lindholm et al., 2015 [34] investigated the effects of rTMS delivered to S1/M1 and S2. However, there is no report on the comparison between M1/S1-rTMS and sham, which does not permit a proper interpretation of their results. Another important feature that deserves special attention is that the targeted cortical region used by Lindholm et al., 2015 [34] differs from the classical site of stimulation used for pain relief (e.g. M1). Lindholm et al., 2015 [34] found a significant pain decrease when the stimulation was performed on S2, compared to S1/ M1 and a placebo. These are intriguing findings, since they oppose the results reported in the early studies published by Tsubokawa et al. [15, 16] who reported better results with motor cortex stimulation in the treatment of deafferentation pain secondary to central nervous system lesions, compared to stimulation of other brain regions. The use of S2 as a cortical rTMS target can also be considered unusual, at least in pain research. For example, in a broader systematic review with meta-analysis, O'Connell et al., 2018 [49] investigated the efficacy of TMS and tDCS used for chronic pain treatment. Even comprising chronic pain of all origins, the study by O'Connell et al., 2018 [49] included only two articles that used S2 rTMS to treat chronic pain. Noteworthy, Lindholm et al., 2015 [34] included patients with three different diagnoses (Table 4), each characterized by specific mechanisms and possibly different responses to treatment. The internal validity of the study was also compromised by important limitations in the methods of randomization, concealed allocation, and blinding of both participants and researchers (Fig 2). Furthermore, this was also the only study evaluating the effects of rTMS on pain that adopted a crossover design. Thus, despite a relatively long wash-out period, a carryover effect cannot be completely ruled out.

Only the study by Umezaki et al., 2016 [48] evaluated the therapeutic effects of DLPFC-rTMS. In fact, stimulation of the DLPFC has been used in psychiatric research for several years. The positive results achieved with such conditions, including depression, with neuromechanisms recently explored [50], along with the well-documented relationship between chronic pain and other psychiatric disorders [51] encouraged the use of the DLPFC as a target for treatment of chronic pain [52]. Using rTMS, Umezaki et al., 2016 [48] found a significant difference in pain intensity between the active and placebo groups in a sample of BMS patients, without finding concurrent changes in the affective dimension of pain and mood. According to the authors, those findings suggest that the analgesic and antidepressant effects of rTMS may act independently. Umezaki et al., 2016 [48] also speculated that by acting through inhibitory pathways that arise from the DLPFC, rTMS could modulate a possible dysfunction within the limbic system of BMS patients. Despite the promising results obtained by Umezaki et al., 2016 [48], important limitations regarding concealed allocation, blinding of both participants and researchers and the presence of attrition bias limit further conclusions. The small number of participants was an additional limitation of that study.

All rTMS studies were classified as having a high risk of bias in at least two domains of the risk-of-bias assessment. Blinding of sham rTMS is particularly challenging, especially because of the sound, visual and sensory characteristics of the active stimulation. For example, Khedr et al., 2005 [35] elevated and angled the coil away from the head of the subject to apply sham rTMS, while Umezaki et al., 2016 [48] used ECT electrodes under the coil. This method seems to be effective in simulating the visual appearance of active rTMS, although it apparently fails to reproduce the sound characteristics related to rTMS. In the current systematic review, only the sham method used by Galhardoni et al., 2014 [30] was considered satisfactory. In this study, the coil used for sham stimulation closely resembled the coil used for active rTMS. The unique feature of such method was the presence of a shield that was not able to block the magnetic field from passing through it. Thus, that method of sham TMS may have mimicked both the visual and the sound aspects of active TMS, though the sensations produced were likely not similar to those produced by real stimulation.

Sample size is another important feature that must always be evaluated. An inadequate number of participants in a study may contribute to errors during the detection of group differences [53]. Among all TMS studies included in the current systematic review, Galhardoni et al., 2014 [30] had the largest sample 33 participants), while Lindholm et al., 2015 [34]; 2016 [33] had the smallest. Both authors reported a sample size calculation which was always estimated at 20 patients. Nonetheless, despite the sample size calculation, Lindholm et al., 2015 [34]; 2016 [33] did not evaluate the total number of participants indicated in the sample size calculation (n = 20). Instead, at the end of the study, the authors only included a total of 16 patients, which might have increased the probability of type I error (false positive).

In sum, only one author did not find efficacy of rTMS in pain relief when compared to a placebo. Despite the differences among the cortical areas stimulated, the therapeutic protocols used, the pain syndrome evaluated and the stimulation parameters, according the results of the current systematic review, rTMS is a promising approach for the treatment of orofacial pain. This information differs from the results of two recent systematic reviews in the neuromodulation field. Hou et al., 2016 [54] and O'Connell et al., 2018 [55] evaluated the efficacy of TMS and tDCS in fibromyalgia and in chronic pain, respectively. Hou et al., 2016 [54] concluded that M1-rTMS appears to be more effective in pain relief than DLPFC stimulation. On the other hand, O'Connell et al., 2018 [55] reported that high-frequency M1-rTMS as well as tDCS may be effective for chronic pain treatment, although with a very low level of evidence. In addition, they found no evidence that low-frequency TMS or DLPFC TMS is effective for chronic pain. O'Connell et al., 2018 [55] advised that the presence of important sources of bias in the articles evaluated may have directly affected the results of their systematic review and meta-analysis. The apparent divergence between the results of the present study and the results found by Hou et al., 2016 [54] and O'Connell et al., 2018 [55] could be explained by the small number of studies included in the present systematic review, with small numbers of participants recruited, in addition to the high risk of biases of the studies. However, it is important to consider the possible presence of different mechanisms related to the anatomical location (orofacial pain versus pain in other anatomical segments) as well as the type of pain (e.g. neuropathic versus nociceptive pain).

The still scarce information regarding the mechanisms related to TMS and tDCS-induced analgesia also affects the proper standardization of the therapeutic protocols designed for its use. It is important to better understand the distribution of the electrical current through the cortical and subcortical structures, and whether it significantly affects the clinical findings of TMS and tDCS studies. Previous studies indicated that M1-rTMS as well as DLPFC-rTMS may increase the activity of deeper brain structures such as the periaqueductal gray matter (PAG) and the anterior cingulate cortex (ACC), The activation of these remote structures, far from the stimulated cortical area, may be related to the recruitment of several neural pathways [56, 57]. It has also been reported that the degree of penetration of the current depends on the stimulation intensity, which in turn is determined by the resting motor threshold (RMT) [56]. The RMT represents the smallest rTMS stimulus that produces a motor response. It is also a method to evaluate the motor cortex excitability. However, the correlation between motor cortex excitability and the activity of other cortical regions has not been completely clarified [56].

Despite the heterogeneity among the studies that evaluate TMS, the motor cortex was still the cortical preferred target and high frequency stimulation was used in all TMS included studies (e.g. 10hz has been the frequency used in 3 out of the 4 studies). All articles that evaluated the effects of TMS, applied this method in neuropathic pain patients. In addition the majority of the studies employed a multi-session strategy.

Regarding tDCS studies, only Brandão Filho et al., 2015 [29] have explored the effects of cathodal DLPFC stimulation. The study by Brandão Filho et al., 2015 [29] was also the only included study that explored the effectiveness of a single tDCS session. The authors did not find differences in the reduction of pain-intensity among the three groups analyzed: sham, 1mA and 2 mA cathodal DLPFC-tDCS. They also reported no differences between the baseline and post-treatment values. In fact, as previously described, DLPFC is a potential target for brain stimulation, although the precise mechanisms and pathways that underlie the analgesic effects driven by DLPFC stimulation are still unclear. In addition, cathodal stimulation, the approach adopted by Brandão Filho et al., 2015 [29], is not as common as anodal stimulation for pain treatment. It is well known that the cathode electrode causes hyperpolarization, thus decreasing the excitability of the neuronal membrane [56]. The intensity at which the electrical current is delivered also has been discussed when cathodal stimulation is applied, with no consensus regarding the most appropriate intensity to use. This interesting topic has been discussed in several articles. According to some authors, either anodal or 2 mA cathodal stimulation increases the cortical excitability, whereas 1 mA stimulation may result in more-specific effects (e.g. at 1 mA, cathodal stimulation seems to decrease the cortical excitability) [58]. To address all these questions, Brandão Filho et al., 2015 [29] conducted an RCT to investigate the effects of cathode tDCS at different intensities. Nonetheless, the authors suggested that this mechanism needs to be to further explored by studies that compare anodic and cathodic DLPFC-tDCS. The single-session treatment may also have compromised the reported clinical outcomes.

All other tDCS studies evaluated the effects of M1 stimulation on pain treatment. Donnell et al., 2015 [31] and Oliveira et al., 2015 [37] used similar protocols, with differences related to the type of setup and the combination of tDCS with an additional therapy. Donnell et al., 2015 [31] used 2x2 HD (High Definition)-tDCS, while Oliveira et al., 2015 [37] combined tDCS with physical therapy. Oliveira et al., 2015 [37] found a significant difference in the pain decrease produced by both sham and active tDCS when comparing pre- and post-treatment values, but found no significant differences between sham and active tDCS. On the other hand, Donnell et al., 2015 [31] reported a significant difference between active and sham tDCS one month after the end of the treatment. Oliveira et al., 2015 [37] suggested an interesting theory to explain the absence of clinical results in their study involving active tDCS. They postulated that their therapeutic protocol, including therapy with exercises, combined with M1-tDCS might have increased the motor excitability, thus negatively affecting the modulation induced by M1-tDCS. On the other hand, the HD-tDCS setup used by Donnell et al., 2015 [31] may also have contributed to the clinical improvement reported. According to the authors, the centered location of M1 between the anode and cathode electrodes might have enabled a posterior-anterior stimulation of the superficial fibers of the precentral gyrus, which run tangentially to the cortical surface. The differences in the stimulation parameters between the studies of Donnell et al., 2015 [31] and Oliveira et al., 2015 [37] might have impacted the clinical outcomes obtained. Another important factor that might have affected the results was the inadequate personnel blinding in the study by Donnell et al., 2015 [31]. In addition, the authors did not adequately report their results, leading to a high risk of bias. In fact, Oliveira et al., 2015 [37] was the only study included in this systematic review that was considered as having a low risk of bias in all risk-of-bias assessment criteria.

Hagenacker et al., 2014 [32] demonstrated significant difference in pain relief when comparing active and sham M1-tDCS. However, it must be considered that the protocol used in this study had important differences from the protocols used in other tDCS studies evaluated in here, such as the use of self- administered tDCS; the use of an low electrical current, at an intensity of 1 mA; and the larger number of sessions (daily tDCS sessions for over two weeks). Furthermore, this was the only tDCS study that included patients diagnosed with neuropathic orofacial pain (e.g. classic trigeminal neuralgia). The results of this study were compromised by systematic failures in its execution. The study by Hagenacker et al., 2014 [32] had the highest rate of participant losses, which represented a high risk of attrition bias. According to the authors, the relatively high dropout rate may have been caused by the type of protocol chosen, in that case, self-application of tDCS. In addition, the authors did not adequately describe how the randomization, allocation, and blinding of participants and researchers were performed.

Adequate blinding associated with tDCS has been previously discussed. Blinding of both participants and researchers has been considered inadequate at an intensity 2mA. In addition, the risk of inadequate blinding of participants would be greater in crossover protocols or in participants who had been stimulated previously [35]. Personnel blinding has also been questioned at an intensity of 1mA [36, 37]. Nonetheless, the evidence of inadequate blinding related to different intensities of electrical current is still very limited, and therefore this criterion was not included in the present systematic review.

In studies that evaluated the efficacy of tDCS there was less heterogeneity in the treated condition and target stimulation region when compared to articles that evaluated the efficacy of TMS. The myofascial TMD has been the most frequent diagnosis, comprising participants from three out of the four articles included. Anodal M1 tDCS has been the method most used among the studies, preferably in multiple sessions.

## Conclusion

Neuromodulation has recently emerged as an attractive alternative for orofacial pain management. However, its efficacy has not yet been established. The studies included in this systematic review showed wide heterogeneity in their therapeutic protocols. Furthermore, most of them were conducted with a small number of patients and a high risk of biases, thus providing a low quality of evidence. Despite the differences in the cortical areas stimulated, the therapeutic protocols used, the pain syndrome evaluated, and the stimulation parameters, the results of the current systematic review indicate that rTMS is a promising approach for the treatment of orofacial pain. In addition, tDCS applied over M1 may be effective in relieving chronic orofacial pain. The results of this systematic review indicate that further research should be carried out with caution in primary chronic orofacial pain, such TMD, due to the existence of scientific evidence demonstrating the effectiveness of less invasive therapies. On the other hand, more research is needed to scrutinize the efficacy of TMS and tDCS in the treatment of other chronic orofacial pain with more robust scientific evidence. In addition, it is imperative to establish better-standardized therapeutic protocols. Although M1 is the traditional region stimulated for pain treatment, the results reported with S2- and DLPFC-TMS suggest that these regions might be potential neuromodulation targets for future studies of orofacial pain patients.

## Supporting information

S1 Checklist PRISMA checklist for systematic review reports. (DOC)

S1 Table Adverse effects reported in each included article. (DOCX)
