# Supplementary material for: The efficacy of transcranial direct current stimulation and transcranial magnetic stimulation for chronic orofacial pain: A systematic review
Source: PLoS One. 2019 Aug 15;14(8):e0221110. doi: 10.1371/journal.pone.0221110 (PMC6695170; doi:10.1371/journal.pone.0221110)
Supplement: S1 Table — (DOCX) [file pone.0221110.s002.docx]

|  | **Adverse Effects** |
| --- | --- |
| **Khedr et al., 2005** | “No patient experienced adverse effects” |
| **Lindholm et al., 2015** | “No serious adverse effects were found. Active rTMS induced unpleasant contraction of the temporal muscle in two patients whose stimulation intensity was particularly high because of high RMT.” |
| **Galhardoni et al.,2014** | No side effects reported. |
| **Umezaki et al., 2016** | “Though seven patients in the real treatment group and five patients in the sham group complained of headache as a side effect, at the beginning of treatment, this symptom was very mild and disappeared in one or two days. No one explicitly dropped out of the study because of side effects.” |
| **Brandão Filho et al., 2015** | “The volunteers did not present significant adverse effects. All types of stimulation were well tolerated. In the two types of active intervention (1mA and 2mA), the frequent side effect was scalp redness. However, no statistically significant difference between active and sham groups were found.” |
| **Donnel et al., 2015** | “There was a low rate of adverse events during the trial, and when present, side effects were mild. They varied from headache to scalp burn (sensation). However, no skin lesions were observed in the areas adjoining the targeted area. Patients in the active group experienced 3.33 side effects per session while patients in the sham group experienced 3.32 side effects per session. The rates of side effects, only measured in the active group, were: tingling (56.7%), headache (50.0%), scalp pain (45.0%), and a burning sensation (43.3%). This study had a 0.8% occurrence rate of severe side effects in the active group.” |
| **Hagenacker et al., 2014** | “All patients tolerated tDCS well, without adverse events. Usually at the beginning of the stimulation, patients reported the presence of a slight itching or tingling, while no motor symptoms were observed or reported during the stimulation”. |
| **Oliveira et al., 2015** | “One subject suffered burns on the fifth day of the stimulation, due to acne in the supraorbital region. No other side effects reported.” |

S1 Table: Adverse effects of non-invasive neuromodulation for the treatment of Orofacial Pain
